# Predicting Emotional Experiences through Eye-Tracking: A Study of Tourists’ Responses to Traditional Village Landscapes

**DOI:** 10.3390/s24144459

**Published:** 2024-07-10

**Authors:** Feng Ye, Min Yin, Leilei Cao, Shouqian Sun, Xuanzheng Wang

**Affiliations:** 1 School of Design and Art, Communication University of Zhejiang, Hangzhou 314500, China; yefeng@cuz.edu.cn; 2 School of Design, Central Academy of Fine Arts, Beijing 100102, China; wangxuanzheng@cafa.edu.cn; 3 The Innovation Center of Yangtze Delta, Zhejiang University, Jiaxing 314100, China; caoleilei@zju.edu.cn; 4 College of Computer Science and Technology, Zhejiang University, Hangzhou 310027, China; ssq@zju.edu.cn

**Keywords:** eye-tracking, emotional experience, prediction model, traditional village landscapes, tour phases

## Abstract

This study investigates the relationship between eye-tracking metrics and emotional experiences in the context of cultural landscapes and tourism-related visual stimuli. Fifty-three participants were involved in two experiments: forty-three in the data collection phase and ten in the model validation phase. Eye movements were recorded and the data were analyzed to identify correlations between four eye-tracking metrics—average number of saccades (ANS), total dwell fixation (TDF), fixation count (FC), and average pupil dilation (APD)—and 19 distinct emotional experiences, which were subsequently grouped into three categories: positive, neutral, and negative. The study examined the variations in eye-tracking metrics across architectural, historic, economic, and life landscapes, as well as the three primary phases of a tour: entry, core, and departure. Findings revealed that architectural and historic landscapes demanded higher levels of visual and cognitive engagement, especially during the core phase. Stepwise regression analysis identified four key eye-tracking predictors for emotional experiences, enabling the development of a prediction model. This research underscores the effectiveness of eye-tracking technology in capturing and predicting emotional responses to different landscape types, offering valuable insights for optimizing rural tourism environments and enhancing visitors’ emotional experiences.

## 1. Introduction

Traditional villages, as significant cultural heritage tourism destinations, boast rich historical, cultural, and architectural values. However, the rapid growth of tourism in these villages has revealed challenges such as uneven vitality distribution and the need for spatial optimization to enhance visitor experiences [1]. Emotional experiences, derived from cognitive evaluations of natural and humanistic environments, can trigger deep cognitive processing, prosocial behaviors, and enhance cultural identity and heritage conservation [2]. On-site interactions within traditional villages, deeply influenced by the heritage and cultural landscape [3]. The authenticity of cultural landscapes creates memorable experiences, drives positive behavioral intentions [4], and results in tourists’ positive emotions exhibiting an inverted U-shaped pattern. Natural landscapes generally induce relaxation and positivity, while cultural landscapes may trigger excitement, surprise, and curiosity [5,6], highlighting the diverse emotional impacts these environments can have on visitors. Moreover, the design of these landscapes, emphasizing nature–humanity integration, geomantic aesthetics, and ecological wisdom, positively impacts emotional well-being [7].

Despite the significance of emotional experiences in traditional village tourism, evaluating these experiences presents significant challenges due to the multifaceted nature of cultural landscapes and the diverse perceptions of stakeholders. Previous studies have attempted to address this issue by developing sense-based hierarchical assessment frameworks [8], employing eye-tracking experiments to quantify visual perception [9], and utilizing advanced methods like VR panorama and scenic beauty estimation to evaluate the aesthetic value of public spaces [10]. However, there remains a paucity of research focusing on the development of quantitative analysis methods that account for the nuanced interplay between sensory dimensions [11], spatial forms [12], and emotional experiences [13].

Eye-tracking technology has emerged as a valuable tool in tourism research, providing insights into visual attention and engagement, which are closely linked to emotional experiences [14]. It has been used to understand attention distribution and emotional reactions in various environments [15,16,17], but few studies have explored its potential in predicting the emotional experiences of cultural landscapes based on cognitive preferences.

It is important to note that while eye-tracking metrics can be related to tourism experiences, the context in which the study is conducted plays a crucial role. The primary objectives of this research are two-fold: first, to further the application of eye-tracking technology in tourism research, and second, to generate valuable insights that can inform sustainable village development and heritage conservation practices. To achieve these objectives, the use of tourism-related visual stimuli is both justified and essential, as it provides a controlled and relevant context for assessing visitors’ emotional responses. By developing a precise model that captures tourists’ dynamic emotional experiences and unraveling the complex relationship between visual attention, cognitive preferences, and emotional experiences in the cultural landscapes of traditional villages, the proposed prediction model is expected to enhance the accuracy of emotional impact assessments. This, in turn, will contribute to the design of cultural landscapes that evoke positive emotional responses, promote visitor engagement, and ultimately benefit tourism management practices.

## 2. Related Works

Historically, the study of emotion in tourism has relied on subjective methods, such as questionnaires and interviews, with tools like the modified Differential Emotions Scale (mDES) [18] effectively capturing self-reported emotions at various phases of a tourist’s visit [19,20]. However, evaluating and preserving the emotional experiences elicited by the cultural landscapes of traditional villages presents significant challenges. These challenges include a heavy reliance on subjective perceptions [8,21], the dynamic nature of rural culture [22], and difficulties in capturing intangible cultural elements [23]. Current assessment methods lack comprehensive frameworks that integrate both tangible and intangible elements of cultural heritage. Due to the intricacies involved, there is an urgent requirement to establish a quantitative analytical approach for assessing the emotional experiences of tourists. This method would improve the accuracy and relevance of emotional evaluations in tourism studies, leading to a better comprehension of how cultural landscapes influence tourist behavior and satisfaction.

The eye-mind hypothesis, as articulated in seminal works [24,25], posits a direct correlation between eye movement patterns and cognitive processes, including emotional arousal and valence. This principle has catalyzed the adoption of eye-tracking technology as an essential tool in diverse fields such as psychology, marketing [26], and human–computer interaction [27]. Eye-tracking offers an unobtrusive and objective method to quantify where and when individuals direct their visual attention, thereby providing profound insights into their cognitive and emotional states [28]. Employing this technology, researchers can assess attentional patterns [29], enhance the effectiveness of content and user interfaces [30], and delve into human emotions and behaviors [31]. Researchers have employed metrics like fixations, saccades, blinks, and pupil size to study the manifestation of emotional processes through eye movements and pupillary responses [17]. While discussing common eye-tracking metrics, fixation count, either over the entire stimuli or within specific areas of interest (AOI), has been used to determine semantic importance and interest towards emotional stimuli [32]. The average number of saccades can be modulated by the emotional valence and arousal level of the stimuli being viewed, which seems particularly sensitive to negative emotional events and expressions [33,34]. Increases in pupil diameter have been observed in response to positive emotional stimuli, presenting a straightforward, non-invasive method to gauge emotional valence using standard camera equipment [35]. Positive emotional stimuli elicit larger pupil diameters and longer fixation durations, while some studies noted that negative emotions lead to more saccades but shorter fixation durations. These findings suggest that different emotional valences may have distinct associations with eye movement patterns [36]. Additionally, data on fixation duration can indicate which aspects of a visual presentation hold the viewer’s attention and trigger emotional responses, be they positive or negative [37].

Eye-tracking technology offers invaluable insights into the complex interplay of landscape, senses, emotions, and behaviors within tourism experiences [38]. Over the past four decades, numerous scientific studies have utilized eye-tracking techniques to evaluate emotional responses by having participants observe and rate landscape photos, thereby gathering and analyzing data on the emotional reactions elicited by these visual stimuli [39]. In the context of traditional villages with cultural landscapes, eye-tracking can reveal how different landscape features capture tourists’ visual attention and influence their overall experience [40,41,42].

Existing research has applied these eye-tracking metrics, combined with machine learning models, to classify general emotional states (arousal, valence, and basic emotions) in response to various visual stimuli, including images and videos [43]. For landscapes and urban green spaces, several studies have explored the relationships between eye movement patterns and perceived restorativeness [44]. However, few have endeavored to develop prediction models that specifically target emotional valence—distinguishing between positive and negative emotional responses [45]. The complexity of these environments, coupled with the dynamic nature of tourism, presents unique challenges that current prediction models may not sufficiently address. This gap underscores the need to more accurately capture and predict the nuanced emotional responses of tourists in these richly varied settings.

## 3. Research Methodology and Experimental Design

Eye-tracking indicators, such as fixation duration, fixation count, and saccadic amplitude, can provide valuable insights into the emotional experience of tourists. However, environmental factors, individual differences, and the nature of the stimuli presented can significantly influence the interpretation of eye-tracking data in relation to tourism experiences. The primary objectives of this research are two-fold: first, to further the application of eye-tracking technology in tourism research, and second, to generate valuable insights that can inform sustainable village development and heritage conservation practices. To achieve these objectives, the use of tourism-related visual stimuli is both justified and essential, as it provides a controlled and relevant context for assessing visitors’ emotional responses. The research aims to address the following question:

How can eye-tracking technology be effectively utilized to quantitatively assess and predict the emotional experiences of Generation Z tourists in response to the cultural landscapes of traditional villages across different tour phases and landscape types?

To comprehensively address this research question, we propose the following sub-questions:

(1) What are the relationships between eye-tracking metrics (APD, ANS, TDF, FC) and tourists’ emotional experiences (positive, neutral, and negative) in the context of traditional village landscapes?
(2) How do the interactions between tour phases (entry, core, and departure) and landscape types (historical, architectural, economic, and life) influence eye-tracking metrics and, consequently, emotional experiences?
(3) Can a prediction model be developed using eye-tracking data and machine learning techniques to accurately capture and predict the dynamic emotional responses of tourists to cultural landscapes, ultimately contributing to sustainable village development and enhanced visitor experiences?

**Figure 1:**
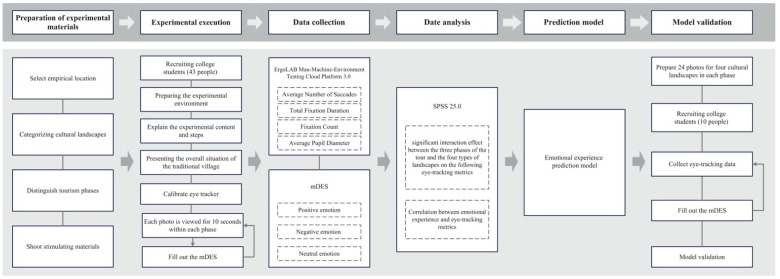
Research framework.

To answer these questions and test the proposed hypotheses, we designed an experimental study (refer to Figure 1) that integrates eye-tracking technology with self-reported emotional assessments using the modified Differential Emotions Scale (mDES) [5]. The study focuses on Generation Z tourists, as they represent a significant and growing segment of the tourism market with distinct preferences and behaviors.

**Figure 2:**
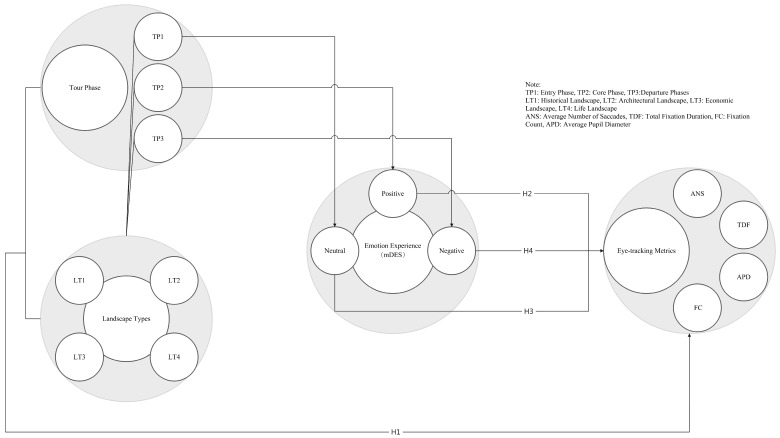
Proposed research model.

The following hypotheses are proposed (refer to Figure 2):

**H1.**  *There is a significant interaction effect between the three phases of the tour and the four types of landscapes on the following eye-tracking metrics: average number of saccades (ANS), total fixation duration (TDF), fixation count (FC), and average pupil diameter (APD).*

**H2.**  *In the core phase of the tour, tourists* *’ positive emotional experiences are positively correlated with eye-tracking metrics (APD, ANS, TDF, FC) across different landscape types (historical, architectural, economic, and life).*

**H3.**  *In the entry phases of the tour, tourists* *’ neutral emotional experiences show no significant correlation with eye-tracking metrics (APD, ANS, TDF, FC) across different landscape types (historical, architectural, economic, and life).*

**H4.**  *In the departure phases of the tour, tourists* *’ negative emotional experiences show significant correlations with eye-tracking metrics (APD, ANS, TDF, FC) across different landscape types (historical, architectural, economic, and life), with the direction of correlation (positive or negative) potentially varying by metric and context.*

### 3.1. Study Area

To accurately assess the significance and correlation of changes in emotional tourism experiences through eye-tracking indicators, selecting tourist destinations with diverse landscapes is essential [46]. The cultural landscapes of traditional villages, like Minhe Village, offer a dynamic and complex setting where architecture, natural scenery, and crowds subtly influence tourists’ emotions [47]. Located in northeastern Wuzhen within Tongxiang City, Zhejiang Province, Minhe Village is characterized by its diverse architectural styles, cultural heritage, farmland, and living scenes that have evolved from the Ming and Qing dynasties to the present. This rich historical context and variety of cultural landscapes across different tour phases make Minhe Village an ideal site for analyzing the variations in emotional experiences and eye-tracking metrics.

### 3.2. Experimental Design

#### 3.2.1. Stimuli

**Figure 3:**
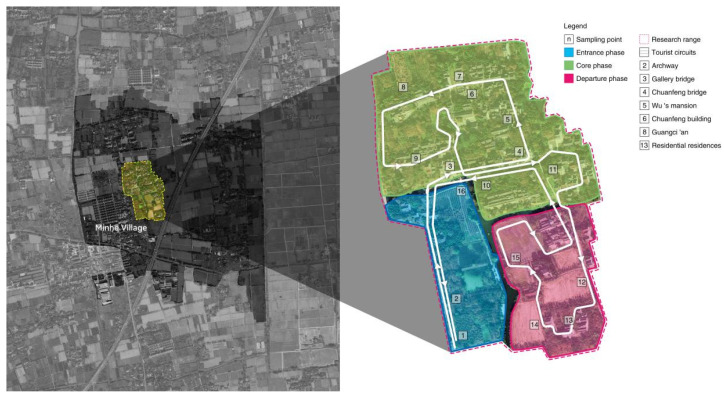
Study area and distribution of sampling points.

To systematically analyze how the eye-tracking metrics related to participants’ emotional experiences vary across different cultural landscapes and tour phases, the study utilizes data collected throughout the tourism experience, as outlined in Figure 3.

In January 2024, the research team visited Minhe Village to capture authentic and representative photos. To ensure consistent visual quality unaffected by varying lighting conditions, photos were taken between 10 AM and 5 PM, optimizing natural light and minimizing shadows or glare. The dataset includes 60 photos depicting historical, architectural, economic, and daily life landscapes of the village, capturing the entry, core, and departure phases of the tourist experience (detailed in Table 1).

Table 1 presents an overview of the landscape types and tourism phases investigated in this study. The pictures displayed are representative examples of each category, selected to illustrate the visual stimuli used in the eye-tracking experiments.

Photos were randomly assigned to either the experiment or validation set [48], ensuring that each set contained an equal number of photos from each landscape type and tour phase.

#### 3.2.2. Participants

The study employed purposive sampling to select a total of 53 undergraduate students from the Communication University of Zhejiang as participants, aligning with the characteristics of the Generation Z tourism group. The participants had an average age of 22 years (SD = 1.2) and a gender ratio of approximately 1:3. They were chosen for their diverse academic backgrounds related to cultural landscape perception, such as design and cultural management. All participants passed a vision test and were naive to the site to avoid preconceived biases.

We selected 43 participants for the data collection experiment and 10 participants for the model validation experiment (53 participants in total). The data collection group provided the primary data for analyzing the relationships between eye-tracking metrics, emotional experiences, tour phases, and landscape types. The model validation group was used to test the prediction model developed based on the data collected from the first group.

#### 3.2.3. Procedure

**Figure 4:**
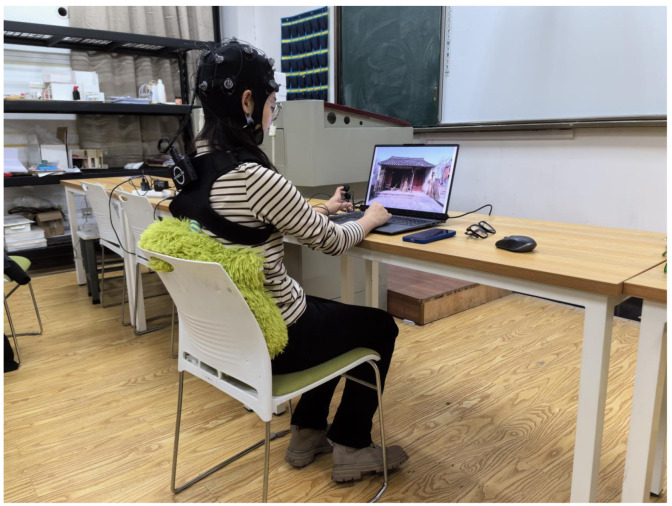
Participants in the eye-tracking experiment using TOBII PRO FUSION.

The experiment took place in a controlled classroom setting to minimize disturbances from noise, odor, and temperature. Participants viewed photos on a 16-inch laptop with a resolution of 2560 × 1600 pixels from a fixed distance of 60–65 cm [49]. Real-time eye movements were recorded using the ErgoLAB Human–Computer Interaction Test Cloud Platform 3.0, coupled with the portable TOBII PRO FUSION from Sweden (Figure 4).

To account for the sensitivity of pupil diameter to changes in brightness, we ensured that all photos were adjusted to have similar overall brightness levels before the experiment [50]. Additionally, during data analysis, we normalized the pupil diameter measurements using a baseline period before each photo was displayed, through the ErgoLAB Human–Computer Interaction Test Cloud Platform 3.0.

In this study, two experiments are conducted: the data collection experiment and the model validation experiment. The data collection experimental procedure was structured as follows: 43 participants first completed a demographic survey. They then underwent calibration with the eye-tracking device to ensure accurate data collection. To mitigate fatigue effects, participants rested with their eyes closed for 3 min before starting the main experiment. During the experiment, each participant viewed 36 photos for 10 s each [49]. A black screen was displayed for 3 s between each photo to minimize carryover effects [51]. The order of the photos was randomized for each participant to control for potential order effects.

To assess participants’ emotional experiences, we employed the modified Differential Emotions Scale (mDES), a widely used self-report measure that captures the intensity of 9 positive (joyful, grateful, amused, content, proud, awed, loving, hopeful, interested), 8 negative (angry, sad, afraid, ashamed, contemptuous, embarrassed, guilty, disgusted) and 2 neutral (surprised, compassionate) emotions on a 5-point Likert scale. The mDES was administered at each phase of the tour (entry, core, and departure) to capture the dynamic nature of emotional experiences.

Participants rated their emotional experiences using the mDES immediately after viewing all the photos from each tourism phase (entry, core, and departure) [52]. The photos were not shown again during the rating process to capture the overall emotional response to each phase [53].

The model validation experiment followed the same procedure, with the 10 participants in the model validation group viewing the remaining 24 photos, covering four types of landscapes across three phases. The data collected from this group was used to test the accuracy and effectiveness of the prediction model developed based on the data from the data collection group.

#### 3.2.4. Data Processing and Analysis

Different eye-tracking metrics can reflect different values of visual attention, emotional arousal/stress and cognitive workload. Four eye-tracking metrics related to emotional valence and arouse [17] were analyzed: average number of saccades (ANS), total fixation duration (TDF), fixation count (FC) and average pupil diameter (APD) (Table 2). The eye-tracking metrics (APD, ANS, TDF, FC) were calculated based on the entire screen area, capturing the participants’ visual attention and emotional responses to the displayed photos [54]. ANS was calculated based on the 10 s viewing period for each photo [55]. After a thorough preliminary check of the raw data, three outliers were removed, leaving 40 valid datasets. Data analysis, including descriptive statistics, variance tests, and correlation analyses, was performed using SPSS 25.0 to explore the relationships between tour phases, cultural landscapes, eye-tracking metrics, and emotional experiences.

## 4. Results

### 4.1. Eye-Tracking Metrics across Different Phases and Landscape Types

To test the H1, we conducted a multivariate analysis of variance (ANOVA) to investigate the impact of excursion phases and landscape types on various eye-tracking metrics: ANS, TDF, FC, and APD.

As shown in Table 3, the interaction between tour phases and landscape types had a significant impact on the average FC (fixation count), F(6, 234) = 2.225, *p* < 0.05, indicating that different combinations of tour phases and landscape types influence the number of times subjects fixate on objects. Additionally, the effect of landscape type on ANS was significant, F(3, 234) = 6.556, *p* < 0.001. The impact of tour phases on TDF, as well as the interaction between tour phases and landscape types on APD, also reached statistical significance (*p* < 0.05). Specifically, FC serves as a critical metric for assessing the demands of visual or cognitive processing.

The results, summarized in Table 3, reveal the significant interactions and main effects, highlighting the complexity of participants’ visual engagement with different landscape types at various tour phases.

### 4.2. Eye-Tracking Metrics in Each Phases and Landscape Types

Further pairwise comparisons, as detailed in Table 4, reveal the effect of the eye-tracking metrics in each phase, as well as the landscape types.

Compared to economic and life landscapes, architectural landscapes elicited significantly more ANS (*p* < 0.01). At the entry stage, both architectural and historic landscapes induced a higher number of ANS than economic and life landscapes. However, during the core stage of the tour, the differences in the number of ANS between various landscape types were not significant, possibly due to a relative balance in the complexity and attraction of the landscapes within the core area of the tour. Finally, at the departure stage, architectural landscapes once again showed a significantly higher number of ANS compared to economic and life landscapes (*p* < 0.05), suggesting that architectural landscapes made a more profound impression on observers towards the end of the tour.

Additionally, analysis of variance for TDF indicates a significant interaction between tour phases and landscape types F(6, 234) = 2.494, *p* < 0.05, ηp2 = 0.06. During the core stage, the TDF on architectural and historic landscapes was significantly longer than that on economic and life landscapes (*p* < 0.05). However, no significant differences in TDF were observed among landscape types during the entry and departure phases of the tour.

Regarding FC, at the core stage, the FC on architectural landscapes was significantly higher than on life landscapes, *p* < 0.05, and the FC on historic landscapes was significantly greater than on economic and life landscapes (*p* < 0.05). APD showed a significant main effect of landscape type, F(3, 117) = 7.46, *p* < 0.001, ηp2 = 0.161. Pairwise comparisons indicated that the APD for architectural landscapes was significantly larger than for economic and life landscapes, *p* < 0.05.

The findings confirm Hypothesis H1, demonstrating a significant interaction effect between the three phases of the tour and the four types of landscapes on the eye-tracking metrics: ANS, TDF, FC, and APD. These results highlight the differential nature of eye-tracking metrics across various tour phases and landscape types, revealing the complex interactions between these variables. Notably, architectural and historic landscapes generally demand more visual and cognitive engagement from tourists, particularly during the core phase of their visit.

### 4.3. Correlation Testing between Eye-Tracking Experiment Data and Emotional Evaluation

To test H2 to H4, we conducted Pearson correlation analyses to calculate the correlation coefficients between the mDES-measured positive, neutral, and negative emotion scores (Supplementary Materials) and each eye-tracking metric (APD, ANS, TDF, FC), revealing their association patterns across different tour phases and landscape types.

The data results (Table 5) indicate that participants’ emotional states significantly influence their visual behavior patterns across different landscape types and tour phases. At the core stage, positive emotions are significantly negatively correlated with the ANS in historic landscapes (r = −0.589, *p* < 0.01), suggesting more stable visual search behavior under positive emotions, and positively correlated with TDF (r = 0.605, *p* < 0.01), indicating prolonged gaze. The data results partially support H2 by showing that, in the core phase of the tour, positive emotions are significantly negatively correlated with ANS and positively correlated with TDF in historic landscapes, indicating that positive emotional experiences influence eye-tracking metrics.

Conversely, positive emotions are negatively correlated with TDF on life landscapes (r = −0.343, *p* < 0.05), reflecting shorter attention spans. In the entry stage, positive emotions correlate positively with FC on life and historic landscapes (life landscapes: r = 0.315, *p* < 0.05; historic landscapes: r = 0.527, *p* < 0.01), due to novelty and curiosity. Positive emotions also increase APD in historic landscapes during the core stage (r = 0.447, *p* < 0.01). For neutral emotions, ANS shows a positive correlation in architectural landscapes during the entry stage (r = 0.348, *p* < 0.05), suggesting more visual exploration, while FC shows negative correlations in architectural and economic landscapes during the core stage (architectural: r = −0.676, *p* < 0.01; economic: r = −0.328, *p* < 0.05). The data results partially support H3 by demonstrating that, in the entry phase of the tour, neutral emotions show a significant positive correlation with ANS in architectural landscapes, indicating increased visual exploration.

During the departure stage, FC on economic landscapes shows a negative correlation (r = −0.395, *p* < 0.05). Negative emotions correlate positively with ANS in economic landscapes during the core stage (r = 0.534, *p* < 0.001) and TDF during the entry stage (r = 0.384, *p* < 0.05), but negatively with FC on historic and economic landscapes during the core stage (historic: r = −0.322, *p* < 0.05; economic: r = −0.648, *p* < 0.001). Negative emotions also correlate negatively with APD in economic landscapes during the core and departure phases (core: r = −0.449, *p* < 0.01; departure: r = −0.329, *p* < 0.05).

The results partially support H4 by demonstrating significant negative correlations between negative emotional experiences and eye-tracking metrics (FC, APD) in economic landscapes during the departure phase, as well as negative correlations with ANS, TDF, and FC in historic and economic landscapes during the core stage.

### 4.4. Landscape-Specific Emotional Prediction Models

After conducting a thorough correlation analysis and evaluation, it was determined that one particular scale exhibited a strong relationship with emotional scores, allowing for the construction of three distinct models. Thus, a stepwise regression was conducted: Model 1, which pertains to positive emotional scores, is associated with historic landscapes. Model 2 relates to architectural landscapes and neutral emotional scores. Finally, Model 3 connects economic landscapes with negative emotional scores. These models underscore the significant impact that different types of landscapes have on varying emotional responses.

#### 4.4.1. Historic Landscapes and Positive Emotional Prediction

The results presented in Table 6, outline the prediction model for positive emotional scores under historic landscapes (Model (1)) as follows:

(1) $$\begin{array}{cc} Positive\;Emotional & Score\\ & =1.417+kTFD\times 0.505+0.475\times eAPD+kAPD\times 0.444\\ & -kANS\times 0.417 \end{array}$$

e denotes the entry area, k denotes the core area, and l denotes the departure area. Thus, kTFD is the total fixation duration in the core area, eAPD is the APD at the entry stage, kAPD is the average pupil diameter the core stage, and kANS is the average number of saccades at the core stage.

#### 4.4.2. Architectural Landscapes and Neutral Emotional Prediction

Table 7 presents the regression results for the architectural landscape, indicating significant predictors of neutral emotional scores. The intercept starts at 4.904. At the entry stage, the ANS has a coefficient of 0.349 (t = 5.391, *p* < 0.001), suggesting that each unit increase in ANS raises the neutral emotional score by 0.349 units, expressed in Model (2):

(2) $$\begin{array}{cc} Neutral\;Emotional & Score\\ & =4.904+eANS\times 0.349-kFC\times 0.404-lAPD\times 0.471 \end{array}$$

eANS represents the average number of saccades at the entry stage, kFC the number of fixations at the core stage, and lAPD the APD at the departure stage.

The model reveals that multiple fixations in the core stage (potentially representing a deeper engagement or complexity of the landscape) correlate with lower neutral emotional scores. Larger pupil diameters at the departure stage, possibly indicating higher emotional arousal or cognitive load, also correlate with lower neutral emotional scores.

#### 4.4.3. Economic Landscapes and Negative Emotional Prediction

Table 8 shows that in economic landscapes, negative emotional scores are significantly influenced by various eye-tracking metrics. During the core stage, an increase in the average number of saccades is positively correlated with negative emotional scores, whereas an increase in fixation count shows a negative correlation, with a coefficient of −0.443 (t = −13.397, *p* < 0.001). At the departure stage, the APD also negatively correlates with negative emotional scores, with a coefficient of −0.439 (t = −8.211, *p* < 0.001). The Beta values indicate that the core stage’s average number of saccades has a significant positive effect on negative emotional scores, while the fixation count and APD exert stronger negative impacts. Thus, Model (3) can be formulated as follows:

(3) $$\begin{array}{cc} Negative\;Emotional & Score\\ & =5.865+kANS\times 0.44-kFC\times 0.443-lAPD\times 0.439\\ & -kAPD\times 0.478 \end{array}$$

kANS represents the average number of saccades at the core stage, kFC the number of fixations, lAPD the average pupil diameter at the departure stage, and kAPD the APD at the core stage.

These findings suggest that in economic landscapes, participants’ negative emotions are related to their visual processing, with more saccades indicating attention to detail and potentially more positive experiences, while higher fixation counts and larger pupil diameters may be associated with higher cognitive loads or emotional arousal, leading to more negative experiences.

### 4.5. Model Validation Test

External validation was conducted to assess the three models: Historic Landscapes and Positive Emotional Prediction (Model (1)), Architectural Landscapes and Neutral Emotional Prediction (Model (2)), and Economic Landscapes and Negative Emotional Prediction (Model (3)). Ten additional participants were involved, each completing both questionnaire ratings and eye-tracking experiments.

The validation process consisted of several steps: firstly, for each emotional condition, the mean scores were calculated from the questionnaire data and averaged within subjects to obtain the mean emotional scores for each participant. Secondly, the eye-tracking metrics of each participant were input into the multiple regression models to calculate the predicted emotional scores. Thirdly, the predicted scores for each participant were averaged within each model to obtain the mean predicted scores. Fourthly, the difference between the mean emotional scores (from questionnaire data) and the mean predicted scores (from regression models) was calculated for each model.

The results(Table 9) indicate that the difference between the predicted scores from the multiple regression models and the actual questionnaire scores was less than 0.5 for each model. This demonstrates that the previously developed models, which divided the tour into different phases and utilized four selected eye-tracking metrics, have high prediction validity for specific subjective ratings. These findings suggest that using objective eye-tracking data to predict subjective evaluations across different tour phases is a feasible and effective approach.

## 5. Discussion

### 5.1. Advancing Eye-Tracking and Emotion Prediction in Tourism

Previous research on emotional experiences in tourism has predominantly utilized subjective methods, such as questionnaires and interviews [18,19,20], which do not fully integrate the tangible and intangible aspects of cultural heritage [8,21]. This study addresses these limitations by combining eye-tracking technology with self-reported emotional assessments to develop prediction models. However, due to the distinct relationships each model highlights between specific types of landscapes and emotional responses, combining the models is neither necessary nor advisable. Each model provides unique and valuable insights, and merging them could obscure these specific findings, reducing the clarity and precision of the results.

Our findings indicate that eye-tracking metrics—ANS, TDF, FC, and APD—are effective predictors of emotional experiences throughout different stages of a tourist visit, especially in interactions with diverse cultural landscapes. This supports the eye-mind hypothesis [24,25] and expands its applicability to cultural tourism contexts. The prediction models formulated in this research enhance the use of eye-tracking technology in tourism and heritage conservation, offering fresh insights into participants’ emotional experiences across different cultural settings.

Additionally, our results show that architectural and historical landscapes require more visual and cognitive engagement from tourists, particularly during the core phase of their visit. This insight adds to the ongoing discourse regarding the role of heritage activities in rural tourism [56,57] and underscores the importance of considering the temporal dynamics of emotional experiences in tourism [58].

By utilizing eye-tracking metrics and prediction modeling, researchers and practitioners can gain deeper insights into how tourists perceive and interact with various landscape elements. This knowledge aids in creating more engaging and emotionally resonant cultural tourism experiences. Ultimately, this study lays the groundwork for the development of evidence-based design strategies that prioritize participants’ emotional well-being and satisfaction, while promoting the sustainable development of cultural heritage sites [59,60,61].

### 5.2. Implications for Cultural Landscape Planning and Tourism Design

Our research provides actionable insights for cultural landscape planning and tourism design, with a specific focus on Minhe Village. The findings emphasize that improving participants’ emotional experiences necessitates a strategic approach that takes into account the distinct characteristics of various landscape types and the temporal dynamics of the tourist journey.

This study reveals the associations between tourists’ emotional experiences and eye movement patterns, particularly the differences in fixation duration and pupil diameter between positive and negative emotions, extending the perspectives of environmental psychology and the assessment of tourism experience quality. The predictive models formulated in this study can help destination managers to develop targeted strategies that enhance the emotional resonance of cultural tourism experiences [46]. Our research provides actionable insights for cultural landscape planning and tourism design, with a specific focus on Minhe Village. The findings emphasize that improving participants’ emotional experiences necessitates a strategic approach that takes into account the distinct characteristics of various landscape types and the temporal dynamics of the tourist journey. Future research could further explore the causal relationships between eye-tracking metrics and emotions, develop more precise emotion prediction models, and provide a scientific basis for landscape optimization.

### 5.3. Limitations and Future Research Directions

While this study offers significant insights, it also presents certain limitations that should be acknowledged. The use of a screen-based eye-tracking system, such as the TOBII PRO FUSION, may not fully capture the immersive and multisensory nature of real-world tourism experiences. The absence of additional sensory inputs and the controlled laboratory setting may limit the ecological validity of our findings. Moreover, the modified Differential Emotions Scale (mDES), though effective for capturing emotional responses, may not adequately reflect real-time emotional fluctuations [62,63].

To address these limitations, future research should explore the use of more naturalistic eye-tracking technologies, such as mobile eye-tracking glasses like Pupil Invisible, in real-world tourism settings. These advanced technologies offer several advantages, including calibration-free design and non-invasive form factor, which would enable researchers to collect eye-tracking data in more naturalistic settings. Conducting comparison studies between screen-based eye trackers and wearable devices in real-world environments will provide valuable insights and contribute to a deeper understanding of the complex interplay between sensory dimensions, spatial forms, and emotional responses in tourism. Future research could benefit from the integration of portable eye-tracking systems with high-resolution physiological signals, such as EEG, to enable more comprehensive, multidimensional assessments.

The prediction model developed in this study should be considered a preliminary model that requires further validation and refinement, using data from additional places and a wider range of participants. As the data were collected from a single location and a relatively homogeneous sample, the correlations and predictive model may not be representative of a wider population or different tourism contexts. Subsequent studies should also aim to broaden their scope by including a more diverse range of case studies, expanding sample sizes, and diversifying sample types to encompass varied landscape elements and tourist demographics [64]. Future research should aim to validate and refine the model using data from additional places and a more diverse range of participants to improve its robustness and applicability.

Additionally, future research should investigate the effects of landscape type density and different phases of the tourist journey on emotional responses. Exploring how interactive and immersive technologies can enhance cultural immersion could also offer valuable insights into improving the design and management of traditional village tourism. By adopting a more holistic approach that incorporates multiple sensory modalities and considers the social and contextual factors shaping tourism experiences, researchers can develop a deeper understanding of the complex interplay between sensory dimensions, spatial forms, and emotional responses in tourism. These advancements could help create more engaging and emotionally resonant tourism experiences, tailored to the needs and expectations of diverse tourist populations.

## 6. Conclusions

This study significantly enhances our understanding of the intricate relationships between eye-tracking metrics, emotional experiences, and landscape types in the cultural landscapes of traditional villages. The prediction models developed herein notably advance the application of eye-tracking technology in the fields of tourism and heritage conservation.

Our findings reveal a substantial interaction effect between tour phases and landscape types on eye-tracking metrics, highlighting how participants’ emotional states profoundly influence their visual behavior patterns. These insights offer crucial guidance for more effective management and design strategies in cultural landscapes.

The practical implications of this research are manifold. They enable destination managers to implement targeted interventions that amplify the emotional impact of cultural tourism experiences, thereby promoting sustainable development. By advancing the use of eye-tracking technology in tourism and heritage conservation, this study lays the groundwork for evidence-based design strategies. These strategies are designed to prioritize the emotional well-being of tourists and support the sustainable development of cultural heritage sites, ensuring that these locales can be enjoyed by future generations while retaining their cultural integrity and historical significance.

## Figures and Tables

**Table 1:** Classification of experimental stimulus materials (examples).

	Entry Phase	Core Phase	Departure Phases
Historical Landscape	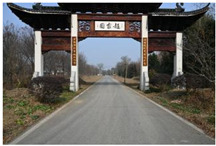	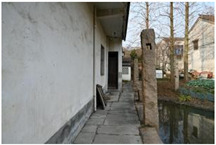	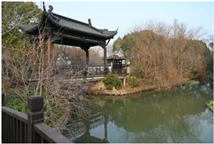
Architectural Landscape	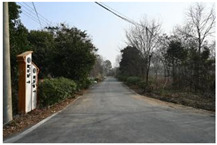	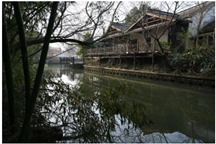	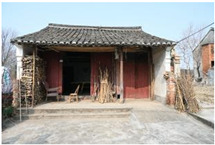
Economic Landscape	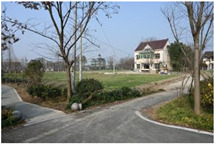	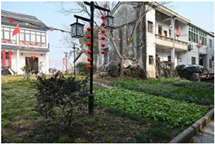	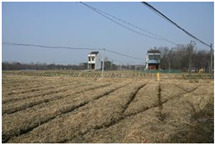
Life Landscape	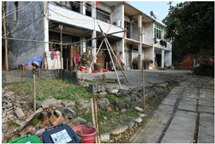	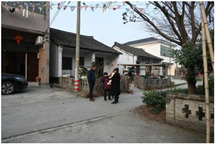	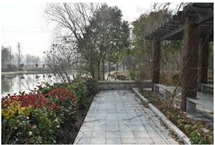

**Table 2:** Meaning of the eye-tracking metrics.

Eye-Tracking Metrics	Meaning
Average Number of Saccades (ANS)	The number of times the observer gazes during a specific period of time. Generally, a higher number of gazes may indicate higher visual or cognitive processing demands.
Total Fixation Duration (TDF)	Total time observed by the observer. Reflects greater attractiveness or richer information for the observer.
Fixation Count (FC)	The number of times the observer gazes. Multiple gazes may indicate repeated attention from the observer.
APD	It is generally considered as an indicator of emotional arousal and cognitive load. An increase in pupil diameter is usually associated with high levels of emotional arousal or cognitive load.

**Table 3:** Multivariate ANOVA of landscape types and eye-tracking metrics at different tour phases.

Eye-Tracking Metrics	Effect	SS	df	MS	F	*p*	ηp2
ANS	Tour Phase	1.257	2	0.628	0.457	0.635	0.012
	Landscape Type	27.503	3	9.168	6.556	<0.001 ***	0.144
	Tour Phase × Landscape Type	17.721	6	2.954	2.225	0.042 *	0.054
TDF	Tour Phase	0.152	2	0.076	0.756	0.473	0.019
	Landscape Type	0.727	3	0.242	2.362	0.075	0.057
	Tour Phase × Landscape Type	1.769	6	0.295	2.494	0.023 *	0.06
FC	Tour Phase	21.975	2	10.988	4.521	0.014 *	0.104
	Landscape Type	9.495	3	3.165	1.269	0.288	0.032
	Tour Phase × Landscape Type	43.866	6	7.311	2.85	0.011 *	0.068
APD	Tour Phase	2.54	2	1.27	2.268	0.11	0.055
	Landscape Type	13.231	3	4.41	7.46	<0.001 ***	0.161
	Tour Phase × Landscape Type	4.926	6	0.821	1.327	0.246	0.033

Note: *: *p* < 0.05, ***: *p* < 0.001.

**Table 4:** Descriptive results of eye-tracking metrics with different landscape types in each tour phase.

	ANS									TDF								
	Entry Phase			Core Phase			Departure Phase			Entry Phase			Core Phase			Departure Phase		
	M	SD	95%CI	M	SD	95%CI	M	SD	95%CI	M	SD	95%CI	M	SD	95%CI	M	SD	95%CI
Architectural Landscapes	3.18	1.22	2.791–3.57	2.83	1.1	2.474–3.179	3.29	1.03	2.961–3.621	0.60	0.33	0.496–0.709	0.76	0.31	0.656–0.856	0.69	0.36	0.577–0.804
Historical Landscape	3.23	1.27	2.825–3.635	2.56	1.06	2.219–2.896	2.78	1.25	2.383–3.182	0.68	0.39	0.557–0.807	0.78	0.32	0.682–0.884	0.70	0.33	0.593–0.806
Economic Landscape	2.43	1.11	2.074–2.781	2.67	1.31	2.25–3.086	2.51	1.17	2.138–2.888	0.65	0.33	0.544–0.754	0.55	0.27	0.466–0.639	0.67	0.35	0.562–0.787
Life Landscape	2.28	0.96	1.973–2.587	2.68	1.37	2.241–3.121	2.62	1.13	2.254–2.979	0.69	0.27	0.603–0.775	0.51	0.34	0.405–0.62	0.70	0.34	0.592–0.807
	F(6, 234) = 2.225, *p* < 0.05, ηp2 = 0.054									F(6, 234) = 2.494, *p* < 0.05, ηp2 = 0.06								
	**FC**									**APD**								
	**Entry Phase**			**Core Phase**			**Departure Phase**			**Entry Phase**			**Core Phase**			**Departure Phase**		
	**M**	**SD**	**95%CI**	**M**	**SD**	**95%CI**	**M**	**SD**	**95%CI**	**M**	**SD**	**95%CI**	**M**	**SD**	**95%CI**	**M**	**SD**	**95%CI**
Architectural Landscapes	3.39	1.62	2.869–3.908	4.38	1.63	3.857–4.901	3.17	1.35	2.737–3.602	3.04	0.84	2.776–3.311	3.12	0.75	2.879–3.36	2.71	0.76	2.463–2.951
Historical Landscape	3.45	1.64	2.926–3.977	4.49	1.63	3.97–5.014	3.57	1.65	3.043–4.099	2.97	0.71	2.742–3.193	2.89	0.7	2.67–3.12	2.67	0.83	2.402–2.934
Economic Landscape	3.6	1.45	3.136–4.061	3.51	1.42	3.056–3.965	3.64	1.69	3.103–4.186	2.7	0.84	2.429–2.967	2.56	0.77	2.312–2.804	2.49	0.86	2.213–2.764
Life Landscape	3.26	1.77	2.69–3.823	3.31	1.35	2.883–3.746	3.77	1.89	3.164–4.376	2.5	0.73	2.265–2.733	2.54	0.59	2.351–2.726	2.68	0.79	2.43–2.938
	F(6, 234) = 2.85, *p* < 0.05, ηp2 = 0.068									/								

Note: M: Mean, CI: Confidence interval, SD: Standard deviation.

**Table 5:** Correlation analysis of eye-tracking metrics in relation to landscape and tour phase.

		Positive Emotions				Negative Emotions				Neutral Emotions			
	Eye-Tracking Metrics	ANS	TDF	FC	APD	ANS	TDF	FC	APD	ANS	TDF	FC	APD
Entry Phase	Architectural Landscapes	0.041	−0.083	−0.159	0.232	0.348 *	0.179	0.035	−0.044	−0.08	0.028	−0.02	−0.086
	Historic Landscapes	−0.221	0.073	−0.197	0.527 **	−0.08	−0.077	0.044	0.118	−0.183	0.003	0.143	0.033
	Economic Landscapes	−0.07	0.069	−0.062	−0.193	−0.242	0.046	0.198	−0.079	−0.256	0.348 *	0.013	−0.02
	Life Landscapes	0.161	−0.034	0.315 *	0.054	0.109	−0.092	−0.181	−0.021	0.078	−0.086	−0.054	0.21
Core Phase	Architectural Landscapes	−0.053	0.141	−0.175	−0.125	0.035	−0.197	−0.676 **	−0.13	−0.237	−0.18	0.016	−0.218
	Historic Landscapes	−0.589 **	0.605 **	0.147	0.447 **	0.051	0.037	−0.033	0.038	0.082	−0.175	−0.322 *	−0.212
	Economic Landscapes	0.093	−0.048	0.173	−0.023	−0.065	0.198	−0.328 *	−0.022	0.534 **	0.207	−0.648 **	−0.449 **
	Life Landscapes	0.058	−0.343 *	0.078	−0.079	0.08	−0.181	−0.111	−0.249	−0.167	−0.057	−0.196	−0.119
Departure Phase	Architectural Landscapes	−0.149	−0.148	0.097	0.033	0.06	−0.073	0.039	−0.205	0.238	0.212	0.142	−0.243
	Historic Landscapes	−0.268	−0.064	0.279	0.182	0.052	0.112	−0.126	0.267	0.146	−0.277	−0.268	0.085
	Economic Landscapes	−0.179	−0.164	−0.086	−0.02	0.146	−0.095	−0.395 *	0.016	0.226	−0.087	−0.138	−0.329 *
	Life Landscapes	0.069	0.126	0.07	0.066	0.056	−0.027	−0.052	0.087	−0.258	0.06	0.058	0.093

Note: *: *p* < 0.05, ** *p* < 0.01.

**Table 6:** Stepwise regression results for the emotion prediction of historic landscapes.

Stepwise		B	SE B	β	t	*p*
1	Constant	2.346	0.252		9.322	<0.001 ***
	kTFD	1.398	0.298	0.605	4.683	<0.001 ***
2	Constant	3.38	0.348		9.701	<0.001 ***
	kTFD	1.078	0.27	0.466	3.986	<0.001 ***
	*k*ANS	−0.306	0.081	−0.443	−3.787	0.001 **
3	Constant	2.312	0.325		7.122	<0.001 ***
	kTFD	0.735	0.211	0.318	3.479	0.001 ***
	*k*ANS	−0.36	0.061	−0.521	−5.882	<0.001 ***
	*e*APD	0.496	0.09	0.479	5.503	<0.001 ***
4	Constant	1.417	0.213		6.66	<0.001 ***
	kTFD	0.505	0.124	0.219	4.076	<0.001 ***
	*k*ANS	−0.417	0.036	−0.604	−11.702	<0.001 ***
	*e*APD	0.475	0.052	0.459	9.186	<0.001 ***
	*k*APD	0.444	0.051	0.427	8.644	<0.001 ***

Note: ** *p* < 0.01 *** *p* < 0.001.

**Table 7:** Stepwise regression results for the emotion prediction of architectural landscapes.

Stepwise		B	SE B	β	t	*p*
1	Constant	4.602	0.307		14.985	<0.001 ***
	kFC	−0.372	0.066	−0.676	−5.659	<0.001 ***
2	Constant	3.761	0.359		10.467	<0.001 ***
	kFC	−0.379	0.058	−0.688	−6.571	<0.001 ***
	eANS	0.274	0.077	0.37	3.533	0.001 ***
3	Constant	4.904	0.384		12.764	<0.001 ***
	kFC	−0.404	0.047	−0.733	−8.602	<0.001 ***
	eANS	0.349	0.065	0.472	5.391	<0.001 ***
	lAPD	−0.471	0.104	−0.4	−4.544	<0.001 ***

Note: *** *p* < 0.001.

**Table 8:** Stepwise regression results for the emotion prediction of economic landscapes.

**Stepwise**		**B**	**SE B**	**β**	**t**	***p***
1	(Constant)	4.888	0.353		13.863	<0.001 ***
	kFC	−0.489	0.093	−0.648	−5.248	<0.001 ***
2	(Constant)	3.719	0.311		11.953	<0.001 ***
	kFC	−0.497	0.066	−0.659	−7.552	<0.001 ***
	kANS	0.449	0.072	0.546	6.262	<0.001 ***
3	(Constant)	4.719	0.329		14.354	<0.001 ***
	kFC	−0.495	0.053	−0.656	−9.396	<0.001 ***
	kANS	0.449	0.057	0.547	7.836	<0.001 ***
	lAPD	−0.405	0.087	−0.325	−4.661	<0.001 ***
4	(Constant)	5.865	0.25		23.498	<0.001 ***

Note: *** *p* < 0.001.

**Table 9:** Validation results for the emotion prediction models.

Tested Model	Average Scores	Model Predicted Scores	Score Difference
Model (1)	3.412	3.173	0.239
Model (2)	3.417	3.265	0.152
Model (3)	3.521	3.092	0.429

Note: Model (1): Historic Landscapes and Positive Emotional Prediction; Model (2): Architectural Landscapes and Neutral Emotional Prediction; Model (3): Economic Landscapes and Negative Emotional Prediction.

## Data Availability

The original contributions presented in the study are included in the article/supplementary material, further inquiries can be directed to the corresponding author.

## Supplementary Materials

The following supporting information can be downloaded at: https://www.mdpi.com/article/10.3390/s24144459/s1, Table S1: mDES-measured Emotion Scores.
