# Supplementary material for: Predicting Emotional Experiences through Eye-Tracking: A Study of Tourists’ Responses to Traditional Village Landscapes
Source: Sensors (Basel). 2024 Jul 10;24(14):4459. doi: 10.3390/s24144459 (PMC11280763; doi:10.3390/s24144459)
Supplement: Supplementary file 1 [file sensors-24-04459-s001.zip › sensors-3065013-supplementary.pdf]

Table S1: mDES-measured Emotion Scores

Description	N	Min	Max	Mean	Standard Deviation
Entry Phase					
Joyful	40	3	5	3.925	0.72986
Grateful	40	2	5	3.625	0.83781
Amused	40	1	5	3.4	1.0328
Content	40	2	5	3.475	0.90547
Proud	40	2	5	3.2	0.96609
Awed	40	1	5	3.3	1.04268
Loving	40	1	5	3.25	0.98058
Hopeful	40	1	5	3.275	0.93336
Interested	40	1	5	3.35	0.89299
Angry	40	1	5	3.75	1.23517
Sad	40	1	5	3.575	1.31826
Afraid	40	1	5	3.5	1.30089
Ashamed	40	1	5	3.275	1.32021
Contemptuous	40	1	5	3.225	1.3679
Embarrassed	40	1	5	3.225	1.34903
Guilty	40	1	5	3.15	1.42415
Disgusted	40	1	5	3.25	1.29595
Surprised	40	2	5	3.4	1.00766
Compassionate	40	1	5	2.85	1.14466
Core Phase					
Joyful	40	3	5	3.95	0.74936
Grateful	40	3	5	3.8	0.72324
Amused	40	2	5	3.675	0.82858
Content	40	2	5	3.6	0.92819
Proud	40	2	5	3.35	0.94868
Awed	40	1	5	3.425	0.9306
Loving	40	1	5	3.275	0.98677
Hopeful	40	1	5	3.275	0.93336
Interested	40	1	5	3.35	0.89299

Description	N	Min	Max	Mean	Standard Deviation
Angry	40	1	5	3.275	1.08575
Sad	40	1	5	3.075	1.09515
Afraid	40	1	5	2.975	0.94699
Ashamed	40	1	5	2.775	1.02501
Contemptuous	40	1	5	2.7	1.11401
Embarrassed	40	1	5	2.75	1.1266
Guilty	40	1	5	2.55	1.21845
Disgusted	40	1	5	3	1.2195
Surprised	40	1	5	3.075	0.97106
Compassionate	40	1	5	2.625	1.03
Departure Phase					
Joyful	40	3	5	3.875	0.68641
Grateful	40	3	5	3.775	0.73336
Amused	40	1	5	3.35	0.83359
Content	40	2	5	3.45	0.90441
Proud	40	1	5	3.025	0.8912
Awed	40	1	5	3.225	0.94699
Loving	40	1	5	3.2	0.93918
Hopeful	40	1	5	3.225	0.8912
Interested	40	1	5	3.275	0.78406
Angry	40	1	5	3.75	1.23517
Sad	40	1	5	3.575	1.31826
Afraid	40	1	5	3.325	1.24833
Ashamed	40	1	5	3.15	1.31168
Contemptuous	40	1	5	3.025	1.27073
Embarrassed	40	1	5	3.075	1.24833
Guilty	40	1	5	3.025	1.3679
Disgusted	40	1	5	3.1	1.25678
Surprised	40	2	5	3.2	0.88289
Compassionate	40	1	5	2.675	1.0225
